# Priorities for contraceptive method and service delivery attributes among adolescent girls and young women in Kenya: a qualitative study

**DOI:** 10.3389/frph.2024.1360390

**Published:** 2024-05-07

**Authors:** Elizabeth K. Harrington, Brett Hauber, Dismas Congo Ouma, Syovata Kimanthi, Annabell Dollah, Maricianah Onono, Elizabeth A. Bukusi

**Affiliations:** ^1^ Department of OB/GYN, University of Washington, Seattle, WA, United States; ^2^ Department of Global Health, University of Washington, Seattle, WA, United States; ^3^ School of Pharmacy, Comparative Health Outcomes, Policy, and Economics (CHOICE) Institute, University of Washington, Seattle, WA, United States; ^4^ Pfizer, Inc., New York, NY, United States; ^5^ Center for Microbiology Research, Kenya Medical Research Institute, Nairobi, Kenya; ^6^ Center for Clinical Research, Kenya Medical Research Institute, Nairobi, Kenya; ^7^ UW-Kenya, Nairobi, Kenya

**Keywords:** adolescent, sexual and reproductive health, family planning, contraception, adolescent-centered, contraceptive preferences, discrete choice experiment

## Abstract

**Introduction:**

Despite increasing global commitment to meeting the family planning needs of adolescent girls and young women (AGYW), there is limited research on how they prioritize contraceptive method and service delivery characteristics. In this qualitative study, we examine the specific elements that drive the contraceptive choices of Kenyan AGYW, and apply our findings to the development of attributes and levels for a discrete choice experiment (DCE).

**Methods:**

Our four-stage approach included data collection, data reduction, removing inappropriate attributes, and optimizing wording. Between June-October 2021, we conducted in-depth interviews with 30 sexually-active 15–24 year-old AGYW in Kisumu county, Kenya who were non-pregnant and desired to delay pregnancy. Interviews focused on priorities for contraceptive attributes, how AGYW make trade-offs between among these attributes, and the influences of preferences on contraceptive choice. Translated transcripts were qualitatively coded and analyzed with a constant comparative approach to identify key concepts. We developed and iteratively revised a list of attributes and levels, and pre-tested draft DCE choice tasks using cognitive interviews with an additional 15 AGYW to optimize comprehension and relevance.

**Results:**

In-depth interview participants' median age was 18, 70% were current students, and 93% had a primary sexual partner. AGYW named a variety of priorities and preferences related to choosing and accessing contraceptive methods, which we distilled into six key themes: side effects; effectiveness; user control; privacy; source of services; and cost. Bleeding pattern was top of mind for participants; amenorrhea was generally considered an intolerable side effect. Many participants felt more strongly about privacy than effectiveness, though some prioritized duration of use and minimizing chance of pregnancy above other contraceptive characteristics. Most AGYW preferred a clinic setting for access, as they desired contraceptive counseling from a provider, but pharmacies were considered preferable for reasons of privacy. We selected, refined, and pre-tested 7 DCE attributes, each with 2–4 levels.

**Conclusions:**

Identifying AGYW preferences for contraceptive method and service delivery characteristics is essential to developing innovative strategies to meet their unique SRH needs. DCE methods may provide valuable quantitative perspectives to guide and tailor contraceptive counseling and service delivery interventions for AGYW who want to use contraception.

## 1 Introduction

As many countries make and carry out FP2030 commitments, improving adolescent and youth contraceptive access and use is increasingly emphasized in country-level family planning (FP) programming (1). Policy documents and published research frequently cite disproportionately high levels of unmet need for contraception among young people (2, 3), and describe the myriad barriers to meeting their contraceptive needs, such as stigma, biased treatment from health providers, beliefs that contraception is harmful, and restrictive SRH policies (3–5). Despite this wealth of data and widely-acknowledged need to provide youth-friendly and tailored SRH services for adolescents and youth, detailed data on contraceptive characteristics and factors that drive choice and promote method satisfaction among young people are relatively lacking, limiting progress toward improving access to quality, person-centered FP care for underserved youth. Choice of contraceptive method—a preference-sensitive decision (6)—is influenced by one's social context and norms (7, 8), and requires making tradeoffs between more and less desired method and service delivery characteristics. Understanding how adolescent girls and young women (AGYW) prioritize the various elements of method choice, and the tradeoffs they are willing to make, could inform novel approaches to delivering contraceptive care that are grounded in a person-centered framework (9), referring to “care that is respectful of, and responsive to, individual patient preferences, needs, and values.” (10).

Discrete choice experiments (DCE) are being increasingly used in health and policy contexts to elicit stakeholder preferences for health-related programs (11). In a DCE, participants are presented with a series of choice tasks in which they are asked to choose between hypothetical alternatives defined by a set of features, or attributes, which are further characterized by variations called levels (12). Responses are then used to quantitatively evaluate the relative importance of the attributes, and the tradeoffs participants are willing to make among the attributes. While DCEs have been used extensively to evaluate user preferences for multi-purpose HIV and pregnancy-prevention technology (13), their application to adolescent and youth SRH has focused on contraceptive development and provider preferences (14–16).

Attribute and level selection is a critical step in DCE design (17), as DCE validity depends on how the complex landscape of options are encoded into a limited number of attributes and levels (18). To avoid biased results, attributes should include all attributes important to decision-making; be non-overlapping without a dominant impact on the decision; and be plausible, relevant, and capable of being traded in the study context (17). Qualitative research is considered a critical element in attribute selection and overall DCE development (19). In response to poor reporting and a lack of rigor in describing the attribute selection process (20), Helter & Boehler describe a more systematic approach to attribute development consisting of four stages: data collection, data reduction, removal of inappropriate attributes, and optimizing wording (21). In this paper, we explore contraceptive method and service delivery preferences among Kenyan AGYW using qualitative methods, and apply our findings to the development of attributes and levels for a DCE.

## 2 Materials and methods

### 2.1 Study design and setting

The data described in this analysis were collected as part of a larger multi-method qualitative study exploring constructs of sexual and reproductive empowerment among female Kenyan adolescents and young adults (22). Data collection incorporated the present paper's focus area on contraceptive preferences, experiences, and decision-making, which were designed *a priori* to inform DCE attribute and level selection. We drew on Helter & Boehler's four stages of attribute development (data collection, data reduction, removal of inappropriate attributes, wording) (21) to provide a structural framework for our methods.

**Figure 1:**
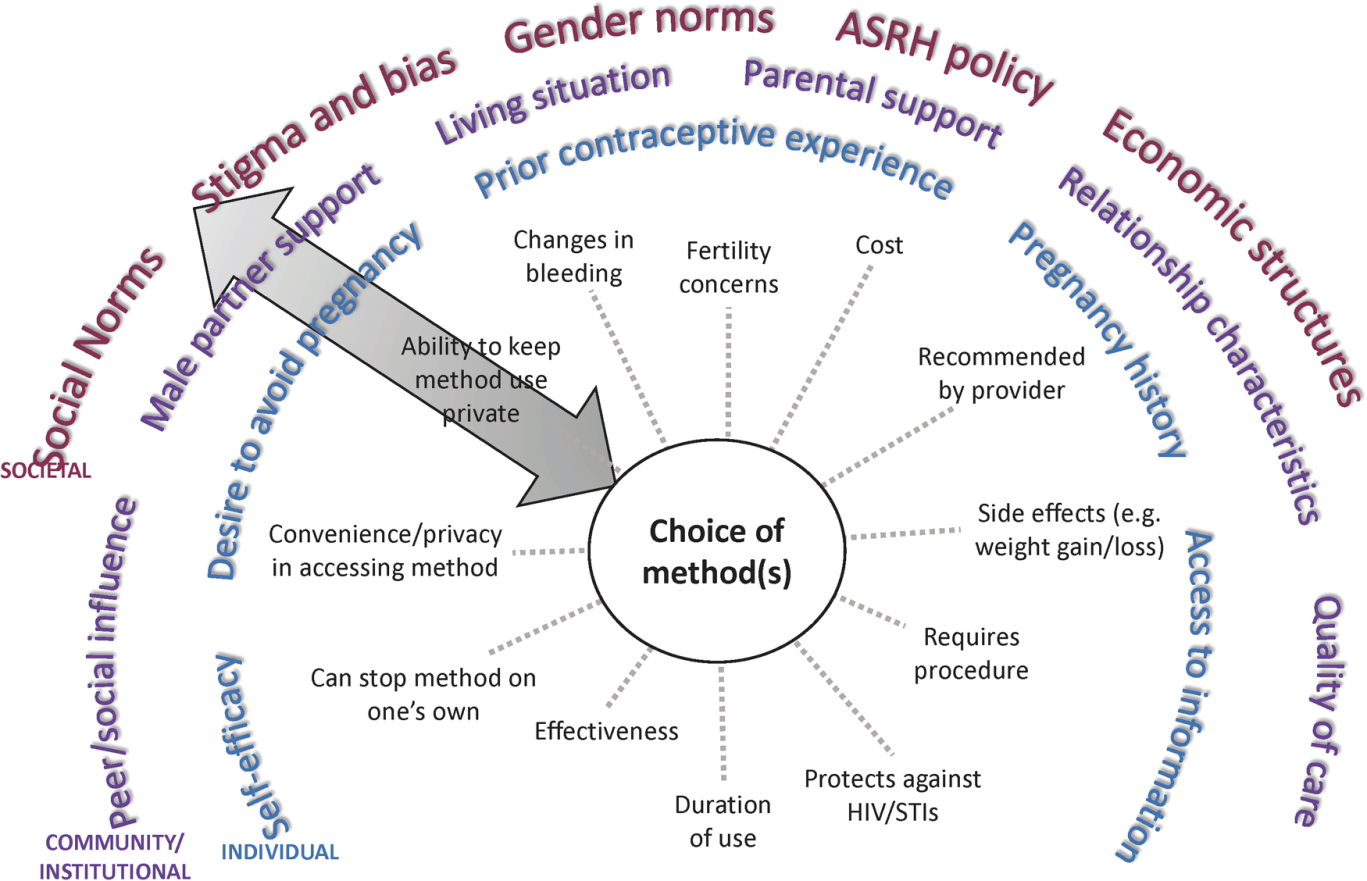
Conceptual map of multi-level influences on contraceptive choice among AGYW. ASRH, Adolescent sexual and reproductive health.

Our approach to the data collection and reduction stages was guided by Brofenbrenner's social ecological theory (23, 24), which is particularly apt in conceptualizing how interpersonal, community, and societal/policy contexts reinforce and influence, and are influenced by, individual-level contraceptive preferences and behaviors. We drew on the social ecological model to create a conceptual map of AGYW contraceptive preferences and their multifaceted influences (Figure 1), which was used to inform the interview guide and eventually to contextualize initial candidate attributes (Supplementary Table). We used qualitative in-depth interviews to gather rich narrative data, followed by structured cognitive interviews for DCE pre-testing and iterative revision.

This study was conducted in urban and peri-urban Kisumu, which is Kenya's third-largest city, bordering Lake Victoria. The predominant ethnic group in the region is the Luo people, and Dholuo and Kiswahili are widely spoken, as is English. We chose to situate the study in Kisumu county for two reasons: (1) our research collaboration's long-term commitment to improving SRH in the region, and (2) as a natural extension of our prior qualitative work in Kisumu and nearby counties indicating a critical need for person-centered contraceptive programs to better serve AGYW within their life contexts (7).

### 2.2 Ethics approval and consent to participate

The study was approved by the Kenya Medical Research Institute (KEMRI) Scientific Ethics Review Unit (P00152/4,193), the Kenya National Commission for Science, Technology, and Innovation (NACOSTI/P/21/10,896), and the University of Washington Human Subjects Division (STUDY001172). Given the overall low risk of the cross-sectional survey procedures and potential invasion of privacy with parental involvement, a waiver of parental permission was approved. Written informed consent (age 18–23) or assent (age 15–17) was obtained for in-depth interview participants; verbal consent was obtained for cognitive interviews. All participants received 500 Kenyan Shillings (approximately USD$4) in appreciation of their time.

### 2.3 Sampling

*In-depth interviews*: Female AGYW were purposively sampled for maximal variation with quotas for age (50% or more age 15–19 years) and prior contraceptive experience (50% or more with prior contraceptive use). Participants were eligible if they were 15–23 years old; spoke Dholuo, Kiswahili, or English; were sexually active in the last year; had the capacity for pregnancy (not currently pregnant or sterilized); and stated a desire to avoid pregnancy for the next 6 months. Eligible participants were recruited in-person in equal numbers from community venues (youth program meetings, markets, informal gathering spaces) and from an ongoing clinical trial cohort based at KEMRI research clinics (25).

*Cognitive interviews*: We used convenience sampling to recruit participants using the above eligibility criteria at KEMRI research clinics.

*Sample size:* The in-depth interview sample size was determined based on research team judgment that 30 interviews would be adequate to generate thematic saturation (26) based on the specific study population and research questions, in line with published guidance (27). Cognitive interview sample size was selected as a reasonable estimate of the number needed to iteratively revise the draft DCE instrument and materials. In-depth interview respondents were not eligible to participate in cognitive interviews.

### 2.4 In-depth interviews *(data collection)*

The research team developed a semi-structured interview guide (Supplementary Material) to explore contraceptive perceptions, desires and preferences, experiences, and decision-making among AGYW, as well as the people and factors at various levels that influence their contraceptive priorities and choices. Interviewers attended a comprehensive training, which included material on research ethics, building rapport with adolescent research participants, research standard operating procedures, and one-on-one practice with the interview guide. We did not conduct pilot testing of the guide, but did iteratively revise the guide throughout the study. Interviewers did not have a prior relationship with participants, and the recruitment and consent/assent processes clearly delineated the researchers and institutions involved, and the goals of the research. Each participant was asked open-ended questions such as: “When you think about choosing a family planning method, what comes to mind for you? What are the most important factors for you when you think about choosing a family planning method?” Subsequently, participants were asked follow-up questions and specific questions relating to candidate attributes (Supplementary Table). All interviews (*n* = 30) were conducted in Dholuo, Kiswahili, and/or English by two Kenyan trilingual interview team members with extensive qualitative data collection experience (including AD). Each interview of 60–75 min' duration was digitally recorded, transcribed, and translated by the interview team.

### 2.5 Data analysis and attribute selection (data reduction, removal of inappropriate attributes)

Data collection and analysis were conducted concurrently. The analytic team (EKH, OC, SK) developed an initial codebook based on topics and concepts in the interview guide and an initial read of several raw transcripts. All transcripts were triple-coded, and the coding team met regularly to revise the codebook with data-specific concepts, review and resolve coding differences, and discuss emerging concepts. The data were analyzed with an inductive, constant-comparative approach (28) to continuously compare and contrast thematic elements within and between participants. Codes were distilled into analytic categories, and the analytic team summarized these categories, or themes, and the relationships between them in memos. Data were coded and managed in Dedoose software (29).

During and subsequent to data analysis, we iteratively revised the candidate attributes and levels in response to our findings. Based on general guidance in the DCE literature, we aimed to include 8 or fewer attributes with 2–4 levels each to avoid respondent fatigue and to follow recommendations for DCE sample size estimation (30). Attributes that were less relevant to participants' lived experiences and contraceptive decisions were removed. The research team then reviewed, discussed, and revised a final attribute and level table.

### 2.6 DCE pre-testing *(wording)*

The research team drafted language explaining the structure of each DCE question, or choice task, and introducing each attribute and level in Dholuo, Kiswahili, and English. A graphic was chosen to represent each attribute level. We then recruited 15 female AGYW meeting eligibility criteria from KEMRI research clinics to participate in structured cognitive interviews. Interviews focused on comprehension and relevance of introductory material, instructions, and attribute and level wording. Participants completed 2–4 draft choice tasks and provided feedback on the structure and graphics. The interview team took detailed notes to record participant perceptions and feedback, which were summarized in debrief reports. The research team iteratively revised the wording and graphics of the final attributes and levels, as well as the introductory language throughout pre-testing (Supplementary Table).

### 2.7 Reflexivity statement

Our binational (Kenya and the United States) team brought a variety of skillsets and perspectives to the current research. The two-person interview team (including AD) who collected all study data are cisgender Kenyan women, trilingual, and have 5–10 years of qualitative data collection and transcription/translation experience. The interview team had no pre-existing relationship with the participants prior to the study. The analytic team included two Kenyan researchers (OC, SK) with Kiswahili fluency and extensive familiarity with the social, economic, and health systems factors that impact Kenyan AGYW. The primary author (EKH) has collaborated on qualitative reproductive health research in western Kenya since 2008, and the second author (BH) is a health economist with extensive experiencing designing and conducting DCEs; both are based in the United States.

## 3 Results

Participant characteristics are summarized in Table 1. In-depth interview participants (*n* = 30) ranged in age from 16–23, with a median age of 18 (IQR 17–19), and 80% were 19 or younger. The majority (70%) were currently attending school, were currently romantically partnered (93%), and had been sexually active in the last month (60%). About half had been pregnant before (53%). The median score on the Desire to Avoid Pregnancy Scale was 3.5 (maximum possible score is 4). All but one participant had ever used a contraceptive method, and 21/30 (70%) reported using a method in the last month. The most frequently used methods were male condoms (11/21) and contraceptive implants (9/21).

**Table 1:** Participant characteristics.

Characteristic, *n* = 30	*n* or median (IQR)
Age, years	18 (17–19), range 16–23
Age group, years	
15–17	13
18–19	11
20–23	6
Educational achievement	
Primary school or less	4
Secondary school incomplete	15
Secondary complete or above	11
Currently a student	21
Currently has romantic partner	28
Age of current partner, years (*n* = 27)	22 (20–26), range 18–38
Source of financial support	
*Self*	4
*Parent/guardian*	23
*Romantic partner*	16
Employment status	
No employment	24
Formal sector employment	4
Informal sector employment	2
Age at sexual debut	15.5 (14–16), range 13–19
Nulligravid	16
Desire to Avoid Pregnancy Scale* score	3.5 (3.1–3.8), range 1.8–4
Ever contraceptive use	29
Used contraceptive method in last month	21
Method used in the last month (can select multiple)	
Oral contraception	4
Injectable contraception	2
Implant	9
Intrauterine device (IUD)	2
Emergency contraception	1
Condoms	11
Condoms plus another method	5

### 3.1 Contraceptive priorities: Key themes

Participants named a variety of priorities and preferences related to choosing and accessing contraceptive methods, and described a variety of interpersonal and community-level influences on the factors that were most important to them. In the following results, we distill the themes into six categories: side effects; effectiveness; user control; privacy; source of services; and cost.

#### 3.1.1 Side effects

Participants cited side effects, particularly related to bleeding pattern, as a major factor in their contraceptive decision-making. Most participants, including several currently using progestin-only methods like injectable contraception and contraceptive implants, described personal or peer experiences of unscheduled or irregular bleeding—frequently referred to as “over bleeding”—as a key factor driving method choice, satisfaction, and discontinuation. When asked to clarify a comment on regular bleeding as a must-have characteristic in a method, a 19 year-old participant who is currently using an implant explained:

*“I would feel bad about it [irregular bleeding], in fact I would have removed this method [implant] by now. It irritates to wear the sanitary towels all the time. You are not clean, and wet all the time…I don’t even like it when I have my normal menses, I hate it so much. When I was still schooling I would experience over bleeding especially during rainy seasons and I was so moody, and so I don’t like it.” (Interview 24, 1 child, non-student)*

A minority of participants, while they didn't prefer irregular bleeding to regular periods, expressed feeling reassured about their health and fertility by experiencing bleeding and being like their peers. For them, lack of bleeding was far more off-putting or concerning than irregular bleeding. Amenorrhea was referred to as “the thing I really dread” (*Interview 20, age 17, no child*)—even worse than difficult to predict bleeding that could affect sexual relationships. As one 19 year-old participant who had recently stopped injectable contraception in favor of condoms and EC put it,

*“Yeah, I think the bleeding is… let’s say you are bleeding and you have family planning, I think that is better because with that you are sure you have had your periods. You know if you have the family planning you get worried, maybe it can block you from having babies. So, if you see the bleeding at least you know you can still get pregnant.” (Interview 19, no child, non-student)*

Preferences around bleeding patterns were further probed by interviewers, who asked all participants if their perspectives would change if a trusted provider explained that it was healthy and normal to have irregular, heavier, or no bleeding while using a particular method. This line of questioning prompted a variety of responses, though more than half stated they would still not find the undesired bleeding pattern acceptable.

Fertility concerns associated with injectable contraception and implants were particularly prevalent in AGYW's communities, sometimes leading to a preference for other methods that were shorter-acting, like condoms, pills, and EC. For example, a 17 year-old secondary school student who discontinued the injectable due to side effects and is now using pills explained how her 24 year-old partner has influenced her method choice:

*“At times [my partner] sits and looks at [my] arm to see if there is something; they [men] like looking at people’s arms to confirm if you have a method. Then he asks, “you haven’t gone for family planning”? When I ask why he…tells me that he doesn’t want me to go for it, he says it will make me fail to have children in future.” (Interview 29, no child, student)*

#### 3.1.2 Effectiveness

Multiple AGYW prioritized how well a method works to prevent pregnancy, in other words, its effectiveness, over other aspects of contraceptive methods. Other participants had limited awareness that effectiveness differs between methods or demonstrated prioritization of other contraceptive characteristics—especially privacy/discreetness. Tradeoffs between effectiveness and side effects were mentioned several times, as described by this 17 year-old participant who is referring to her use, and dissatisfaction with, a contraceptive implant:

*“I used to bleed a lot and suffer from abdominal pains….[Interviewer asks why she still has the implant despite these side effects] I still just don’t want to remove it…(Chuckles)…I just don’t want to remove it because I was told that I can never get pregnant with it.” (Interview 5, 1 child, non-student)*

Participants frequently equated higher effectiveness with longer duration of use. A 22 year-old woman narrated her experience of an undesired pregnancy after emergency contraception (EC) failed, explaining why she chose to get an intrauterine device (IUD) after procuring an abortion:

*“I feel that it [EC] is not as effective as we are made to believe and that is why I conceived after taking it…I want a method that goes for a long period of time like the coil [IUD], Yeah, I prefer that…I just trust it more. The fact that it is meant to last a longer period means that it is more effective.” (Interview 13, no child, student)*

#### 3.1.3 User control: duration of use and ease of discontinuation

While most participants considered duration of use when describing their rationale for preferring a particular contraceptive method or methods, ease of discontinuation as a factor rarely spontaneously came up. For a few AGYW, though, their method choice was strongly related to avoiding fertility or side effect concerns associated with longer-acting methods and methods for which discontinuation requires provider assistance (IUD, implant). Furthermore, perception that choosing a long-acting reversible contraceptive (LARC) method committed one to using the method for as long as its effective life came up multiple times in interviews. Several participants mentioned preferring the “3-year” implant (e.g., Nexplanon) to the “5-year” implant (e.g., Jadelle), both of which are typically available in the region, or preferring a shorter-acting method for this reason:

*“Okay what comes to my mind first, I would prefer Depo [injectable] because I just use it for the time being, then when I am ready to get a child, I can just leave it. If I inject the one at the arm [implant] and maybe I am ready and he wants a child [so] it will be difficult, because maybe you have inserted the five years method [referring to implant with 5 year duration of use] then after one year you want a baby… [you] will have to wait for five years to have a child. So I prefer Depo.” (Interview 19, age 19, no child, non-student)*

Concerns about delayed fertility after discontinuing the method were influential for several participants, including a 21 year-old woman who had recently experienced an undesired pregnancy. After having an abortion, she chose to start pills due to concerns that a longer-acting method like the injectable would make it difficult to get pregnant in the future. She also summarized many participants' reasoning for not wanting to rely on a male-controlled method like male condoms or a method you have to use every time you have sex:

*“Condom is a good idea but then it depends with the partner you are having. In a case when your partner needs a child and you are not of the idea, you know there is no way you are going to convince him to use a condom. Using a condom will depend if you can cooperate with your partner but knowing the partner I have is not going to cooperate—that is why I use pills.” (Interview 27, age 21, no child, student)*

Several participants, particularly those who had chosen a LARC method, cited a preference for longer duration of use >1 year due to convenience, lower cost over time, and peace of mind:

*“…so that I am never worried of the future and I am always sure that I have the method in my body all the time.” (Interview 10, age 19, 1 child, non-student)*

#### 3.1.4 Privacy

The ability to conceal contraceptive use from parents, romantic partners, peers, and other community members was a critical consideration in method choice for all but a few AGYW. The adolescent-specific concern of maintaining privacy in the boarding school setting came up multiple times among participants who were still attending secondary school as a factor that influenced their contraceptive preferences:

*“I chose IUD because for example with pills, I can’t carry them to school because you have to take after every 24 h, what if someone sees me taking them, rumors can spread that I am having an abortion so that is also an embarrassment that I didn’t want to affect me.” (Interview 21, age 19, no child, student)*

The methods AGYW perceived as the most private differed somewhat between individuals, but overall, the contraceptive implant and oral contraception were viewed as less private than injectable contraception and IUD. Many AGYW discussed their worry that the implant would be seen or felt by partners or parents. A 19 year-old woman (*Interview 9, no child, student*) described her experience accessing contraception on a school break, and how she “settled for Depo [injectable]” because she was wearing a short-sleeved dress and knew that others would see the bandage and incision site on her arm if she had an implant placed. This concern outweighed another concern she had, that she would not be able to get subsequent injections on time due to her school schedule. Another participant, a student who lives with her parents and recently discontinued the injectable in favor of oral contraceptives due to bleeding pattern changes, described her priorities in contraceptive decision-making:

*“For [the] implant I feared the pain. But the main reason I didn’t go for an implant was because I didn’t want anybody to see, because you know there are some parents who will notice, so I didn’t want to be in that situation.” (Interview 27, age 21, no child, student)*

While several AGYW portrayed their male partners as supportive of or indifferent to their contraceptive choices, many indicated a preference to keep method use private from partners, as well as detailing experiences of contraceptive coercion:

*“I had already told him [my partner] that I had a method [implant]. He did not like it, he even told me to go have it removed it because if not, then he would terminate the relationship. I then asked him what was that for and yet he also did not want to use condoms each time that he had sex with me…I later told him that I had removed the method. Currently he is not aware that I use. So I always have to try to hide that spot on my body that was used to insert the family planning because he might touch it and that would make us have conflict.” (Interview 15, age 17, 1 child, student)*

#### 3.1.5 Sources of information and services

When they were specifically asked about their preferred sources of contraceptive information and services, AGYW discussed their reasons for choosing a private sector pharmacy or a clinic facility. Among several AGYW who preferred the pharmacy, perceived privacy was top priority, often superseding cost, which was noted to be higher in pharmacies than public facilities.

*“Pharmacy is a bit [more] private because…you just tell them to inject you and they will inject you. But for clinic, if you go to the clinic, you will meet so many people there (laughs) so even if you want to get a family planning and they are there, people will just know that you are going to be injected. But pharmacy once you are inside, people will not know that you have gone for family planning; obviously pharmacy is a place to buy drugs, so I prefer pharmacy.” (Interview 19, age 19, no child, non-student)*

More than half of participants stated a preference for a clinic, mainly due to a desire for contraceptive counseling, but also related to perceived higher quality of care including trained, more trustworthy providers and non-expired contraceptive methods, availability of female providers, and access to other services like HIV and pregnancy testing. In general, AGYW wanted to receive information about recommended methods and potential risks and side effects from a qualified provider prior to initiating a method:

*“I prefer hospitals because they are like careful with what they are doing and they are like they will advise you before you do anything, clinics and chemists [pharmacies] are just there because of money they don’t screen you, they don’t give you advice, they just insert it.” (Interview 23, age 16, no child, student, using implant)*

Another participant expressed concerns that people working in the pharmacies are not “qualified…They are just there for money and they won’t advise you health-wise.” *(Interview 27, age 21, no child, student*)

AGYW's preferred sources of information influencing their contraceptive choices were health providers, but many relied on parents, other family members, and peers for advice as well. Several participants mentioned learning about various methods' risks and benefits from online sources, such as videos:

*“I decided to use the implant because the way I was getting…the advice, it was better because I love watching encouraging videos, so many people were preferring this and even doctors were preferring this. So I was very comfortable…” (Interview 23, age 16, no child, student)*

#### 3.1.6 Cost

Method cost factored into participants’ decision-making, but it was mentioned far less often than side effects, effectiveness, and privacy. Most commonly, cost influenced decision-making in the direction of desiring longer acting methods that would be cheaper over time. Several AGYW contrasted the lower cost of LARC methods with EC, which is often referred to as “P2”:

*“Implant is good, I chose implant because it won’t bother me several times when I don’t have money, I will not buy P2, you know P2 you have to buy it like that morning I could not also afford. I am using an implant because it is always there.” (Interview 23, age 16, no child, student)*

Similarly, other AGYW commented on the need for follow up visits with use of shorter-acting methods, which cost money and time opportunity cost. A 23 year-old who supports herself as a domestic worker explained,

*“One thing that influenced my decision is that…sometimes you don’t have the money for the TCAs [referring to follow up visits for injection] and end up getting pregnant, that is why I like this one [implant] because it stays for long.” (Interview 16, non-student, 6 children)*

### 3.2 Attribute selection and construction of choice tasks

Concurrent data collection and analysis allowed the study team to iteratively assess the most relevant aspects of AGYW contraceptive decision-making and priorities. To minimize cognitive burden to DCE respondents and optimize the precision of preference estimates, our goal was to select fewer than 8 attributes (31). After completing qualitative data analysis, we selected 7 final attributes (Table 2) and removed the other, less appropriate, attributes using a process that combined findings from our qualitative work and multidisciplinary team consensus-building. First, we identified the attributes that were most relevant to AGYW's priorities and experiences, based on the key themes that emerged from the in-depth interviews. In tandem, we dropped 2 attributes (ability to stop method on your own, privacy during method access) that represented concepts or priorities that the majority of participants said were less important to them or were infrequently spontaneously introduced by participants; we also removed an attribute (timing of return to fertility) that overlapped conceptually with another (how long method will last). We chose to include cost in order to allow for calculation of willingness-to-pay. Attribute levels were also iteratively revised during the analytic process, guided by participant perspectives and team discussion.

**Table 2:** Final attributes and levels.

Attribute	Level 1	Level 2	Level 3	Level 4
How your periods may change	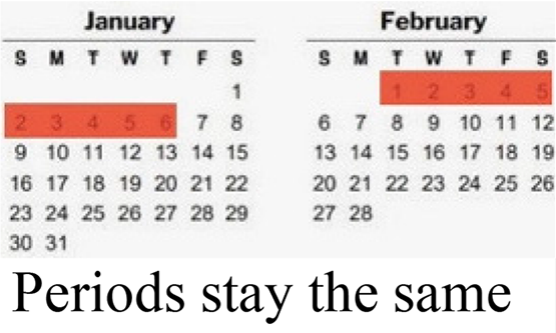	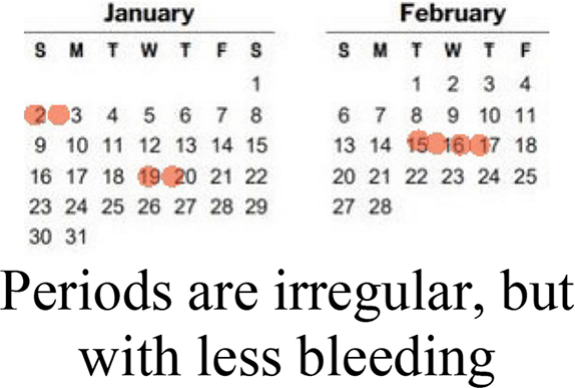	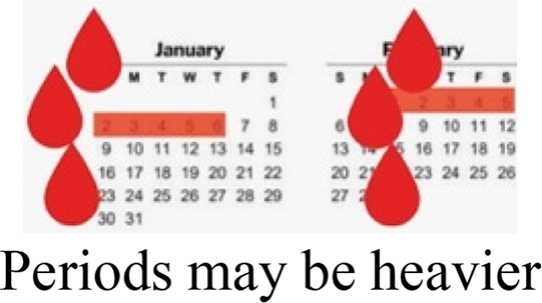	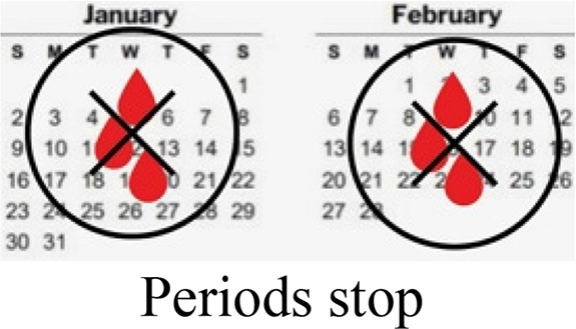
Chance of pregnancy in 1 year	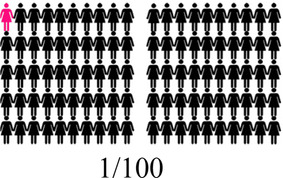	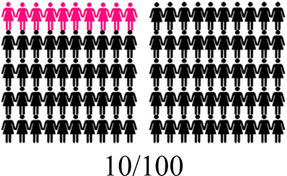	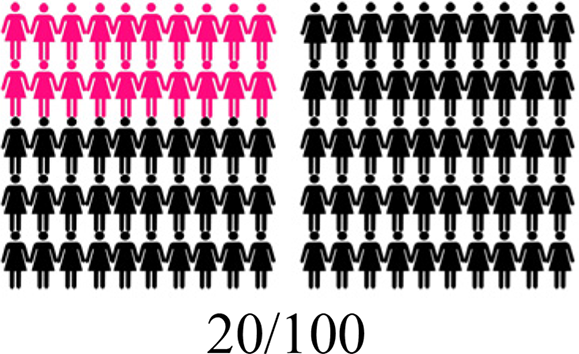	
Ability to keep a method private	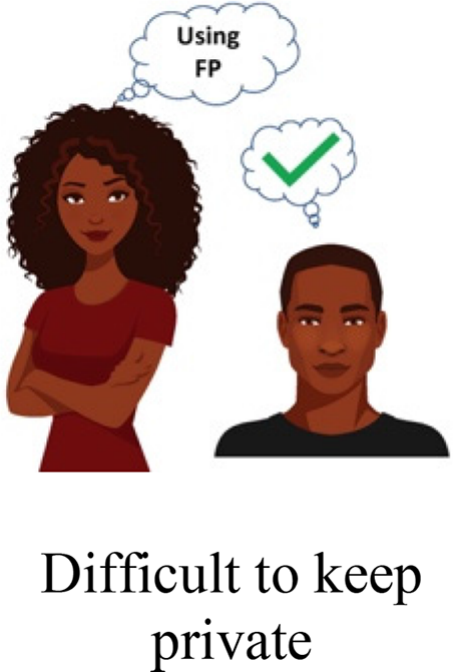	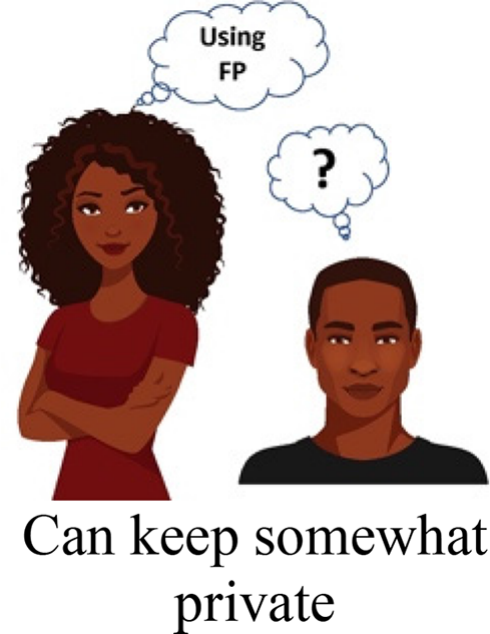	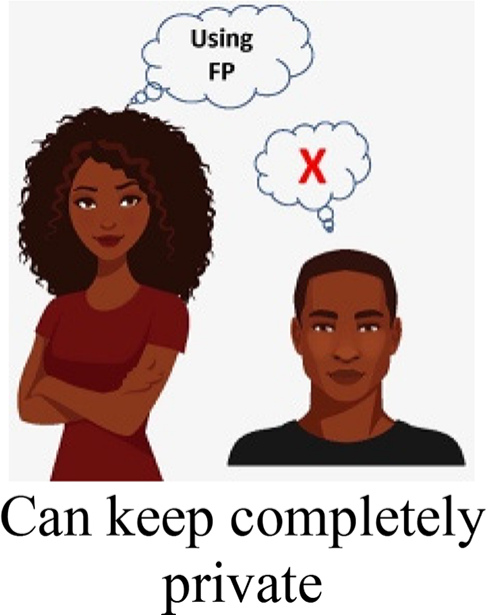	
How long a method will last	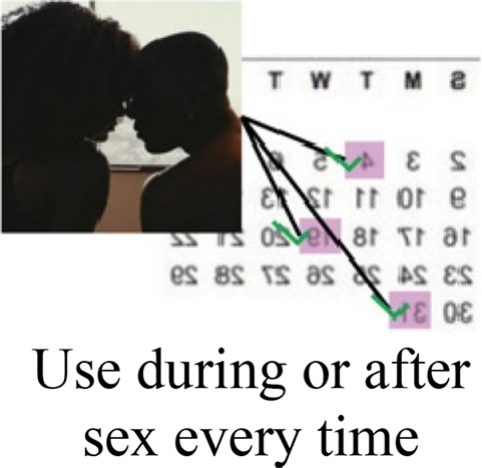	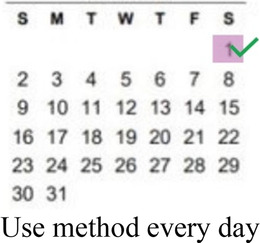	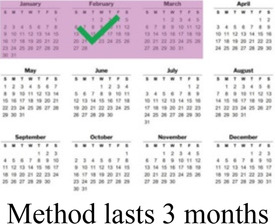	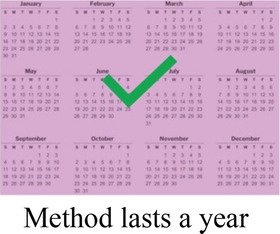
How you will get information about your options	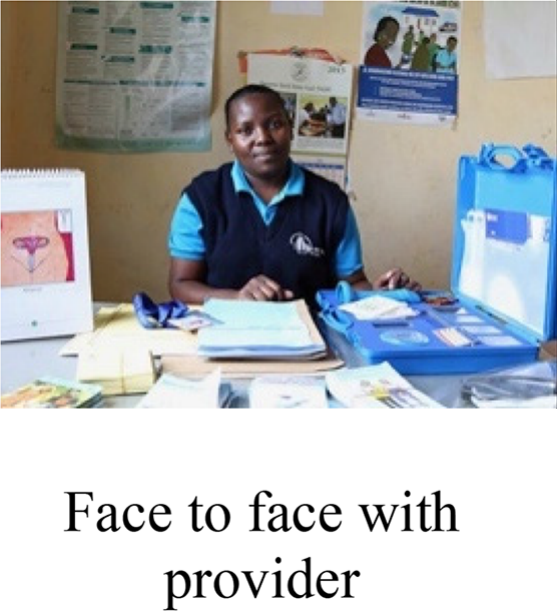	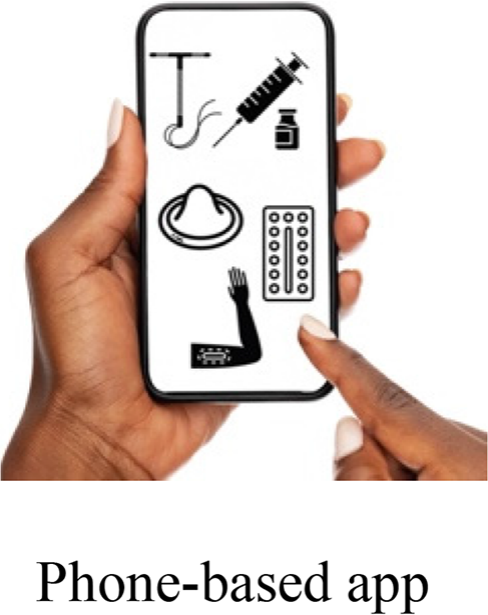	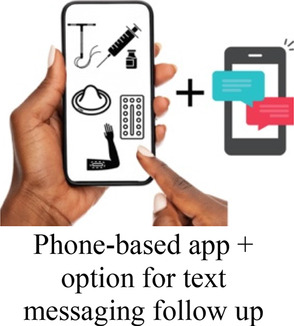	
Location	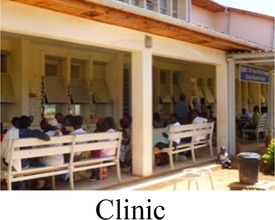	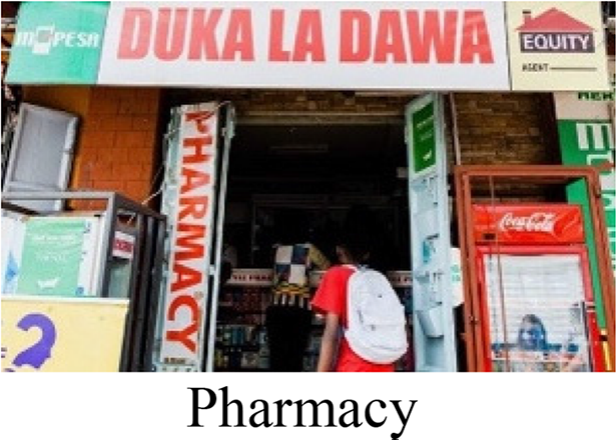		
Cost	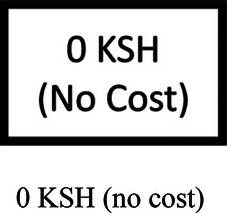	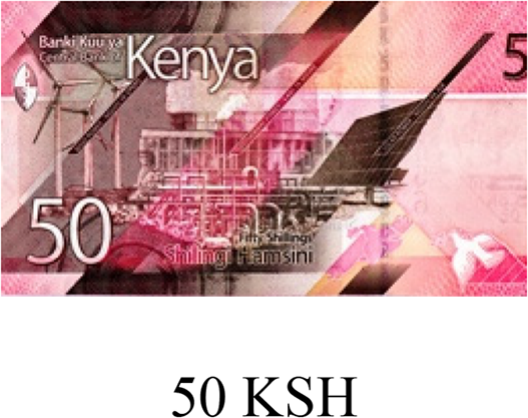	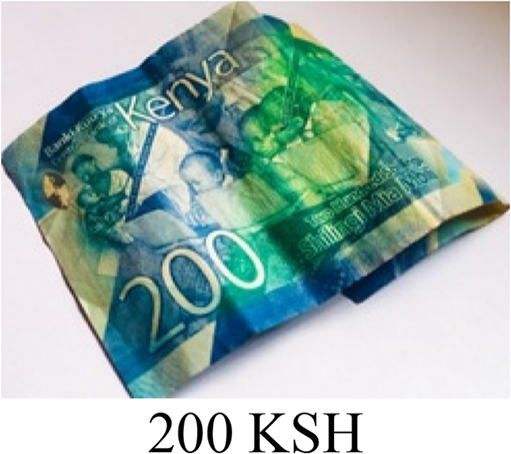	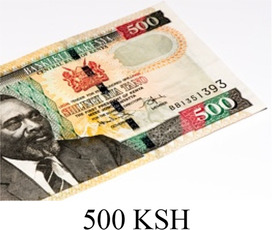

**Figure 2:**
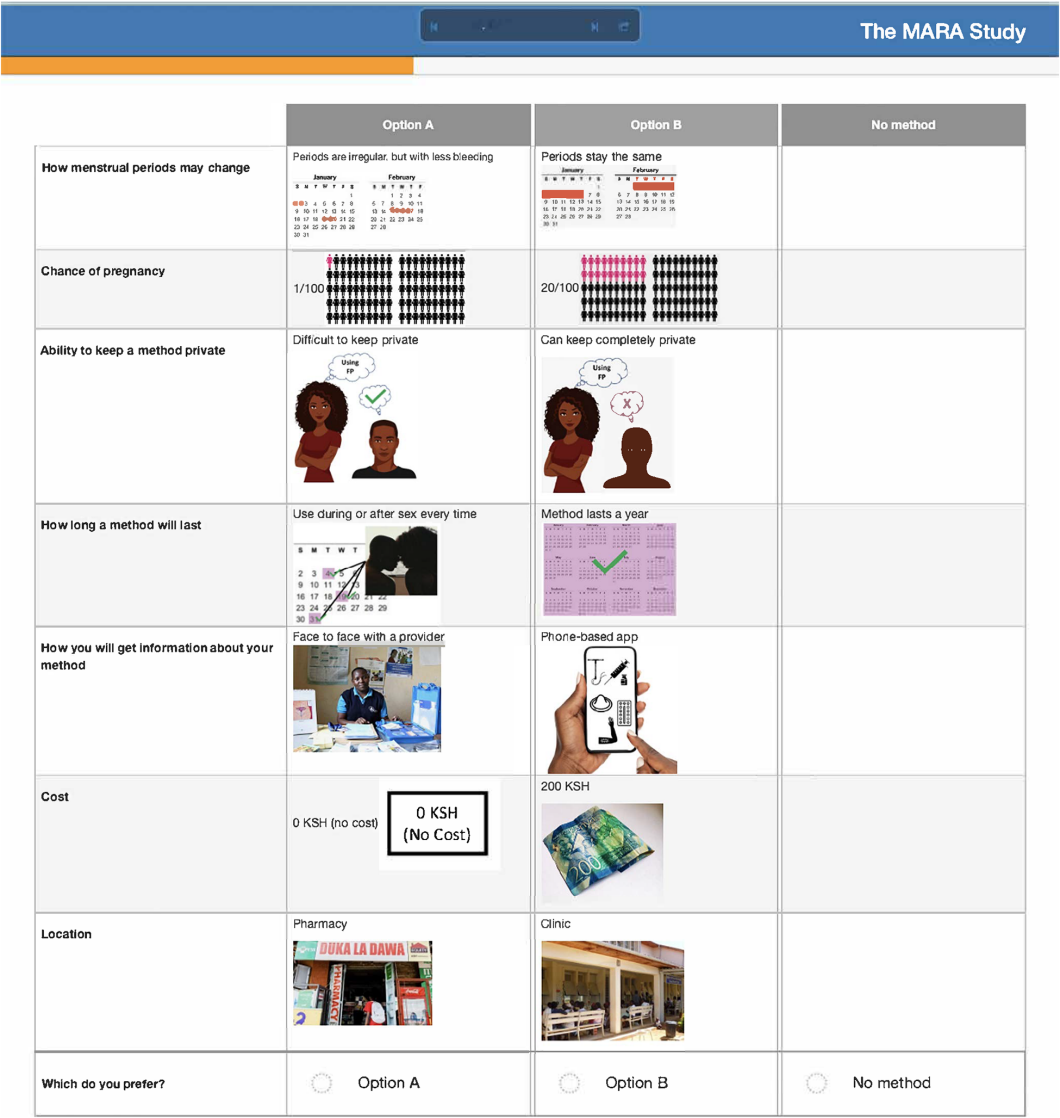
Sample choice task.

After attribute selection, we drew on published guidance (11) to develop the structure of the choice tasks. Choice tasks include two full profiles (labeled “Option A” and “Option B”), meaning that all 7 attributes are represented in each profile, and an opt-out option. We defined the opt-out as “no method” in order to allow participants to indicate they would rather use no method and a higher risk of becoming pregnant than use either hypothetical contraceptive profile A or B. Our qualitative findings provided a rationale for including an opt-out: some participants, despite a strong desire to delay pregnancy, found the tradeoffs associated with contraceptive use unacceptable. Figure 2 presents a sample choice task.

### 3.3 Pre-testing and iterative revision

Participants in cognitive interviews (*n* = 15) had a median age of 21 (IQR 19–23); no other demographic data were collected. Interviewers presented the participants with mock-up introductory material (5 in each language) and 4 example choice tasks, and assessed their understanding of the instructions, eliciting feedback on the clarity of language and translations, initial thoughts related to the images chosen to represent the attribute levels, and comprehension related to how to answer the questions. In response to participant comments and detailed interviewer notes, the team iteratively revised the introductory language, the wording of attributes and levels, and the images. For example, the lowest attribute level for cost was initially labeled as “free”, and the image used was a colorful red shape with the word “FREE” in it. AGYW explained that they chose the option with the “free” image in part because they liked how it looked; to avoid distracting survey participants and introducing bias, we removed the image and labeled that attribute level “0 KSH” (Kenyan shillings). Additionally, we received feedback that the image of a male partner used in the initial sample survey questions was distracting, as AGYW tended to interpret his facial expression as upset or suspicious. This image was replaced with one where the male partner's expression was perceived as more neutral. Finally, we used the cognitive interviews to assess AGYW's reaction to the various attribute levels for cost to establish the most appropriate upper limit of cost. AGYW were willing to choose the profile that cost 500 KSH when the other attribute levels in that profile were favorable, indicating that participants found it reasonable under specific conditions.

## 4 Discussion

This study used qualitative methods to explore what is most important to Kenyan adolescent girls and young women in choosing a contraceptive method, contributing to the limited literature focused on understanding preferences for available contraceptive method and service delivery characteristics in the region. We highlight the factors and tradeoffs that are most influential to these young women's contraceptive choices in the context of their lives and relationships, and apply these qualitative findings to the systematic, iterative process of developing attributes and levels for a DCE. We selected, refined, and pre-tested 7 attributes, each with 2–4 levels, in order to quantitatively evaluate contraceptive method and care preferences and tradeoffs in an upcoming DCE.

Family planning clinicians and programs have frequently emphasized method effectiveness over other features of contraceptive methods, especially for adolescents (32). Globally, there is increasing recognition that directive counseling toward LARC methods can be coercive (33, 34), and that some people value other aspects of a method above its effectiveness—for example, user control or non-contraceptive benefits (9). Our data highlights that minimizing the chance of pregnancy was the primary factor driving some AGYW's method choices, but that many expressed additional priorities that were as or more important—such as bleeding pattern, peer and provider recommendation, and ability to maintain privacy.

Contrary to our expectations, user control, referring to the ability to initiate or discontinue a method on one's own, did not emerge as a key priority for AGYW in this study. The rapid scale-up of contraceptive implants in Kenya and many other countries in Africa over the past decade has significantly altered the method mix (35), and recent data suggest that many implant users face challenges and delays with respect to accessing timely and quality implant removal (36, 37). While AGYW did not focus on concerns about autonomy related to discontinuation, and thus we chose to not select “ability to stop a method on your own” as an attribute, the related concept of duration of use was considered very important. Many AGYW spoke of preferring the “3 year” over the “5 year” method and several believed the method had to remain in place for its entire useful life. This could be due to community-level experiences and narratives of provider reluctance or refusal to remove implants on request. It is also possible that perspectives on the importance of being able to self-discontinue a method would differ among AGYW living in remote rural areas where access to LARC removal is more limited.

Bleeding side effects and changes to menstrual bleeding patterns emerged as a critical element of AGYW's experience influencing method choice, method (dis)satisfaction, and discontinuation. Consistent with other studies from East and Southern Africa (38, 39), amenorrhea was viewed negatively, with irregular or prolonged bleeding undesired, but often tolerated. Prior studies have described how concerns about future fertility are a major deterrent of contraceptive use among many young women (40, 41); our data shows that for some, the presence of bleeding, even if it is irregular, may allay worries that methods will cause infertility.

The decision to not use contraception may be an informed choice (42, 43); additionally, non-use may be related to partner coercion, constraints on access, or other factors, such as feelings of indifference or ambivalence regarding pregnancy. In this study, participants were only eligible if they stated a desire to delay pregnancy for at least 6 months; their median score on the DAP Scale (44) was 3.5 (maximum possible is 4), suggesting a strong desire to avoid pregnancy. However, about a third were not currently using a contraceptive method despite being at risk for pregnancy. In response, and acknowledging increasing interest in understanding the context around non-use, we designed the structure of the choice tasks for the upcoming DCE to include “no method” as an alternative to the two contraceptive method profiles. Including the opt-out option signifies non-use as a reasonable option, even when one does not want to become pregnant at that time, as the choice to use a method relates to the tradeoffs one is willing to make.

Private sector community pharmacies are a critical point of contraceptive access among AGYW in Kenya, often for reasons of privacy and convenience (45, 46). Our findings, consistent with a recent study by Calhoun et al. on young Kenyan women's contraceptive decision-making (47), suggest a high demand for provider contraceptive counseling among AGYW, which steers many to public sector clinics—despite their co-existing needs for privacy and after-hours access. The demand for contraceptive counseling in settings where trained clinicians may not consistently available requires innovative strategies to deliver desired information and advice, which may include pharmacy worker training and digital health interventions. While DCE methods can help elucidate the tradeoffs AGYW are willing to make to get their desired location and services, further implementation research is needed to understand how contraceptive care can be optimized and adapted for more accessible, community-based settings like pharmacies.

A strength of this analysis is the balance we strike in what Coast & Horrocks (2007) refer to as the inherent “tension” between inductive, exploratory qualitative research and the “reductiveness needed to encapsulate the different aspects of a service within a minimum number of attributes” (20). Our findings independently contribute to the limited understanding of AGYW contraceptive priorities, and informed the selection of attributes that reflect the lived realities of the populations this research ultimately aims to benefit. This study also has limitations. While the selected attributes represent the most salient considerations in AGYW's method choices, we recognize that missing key attributes can result in biased DCE findings (17). While our iterative selection process follows published best practices (19, 48), we did remove some candidate attributes or may have missed other factors, which for some AGYW may be important to decision-making. Furthermore, our geographic reach was limited to a single county in Kenya, and like all research using purposive sampling, is not designed to be directly applied to other settings.

## 5 Conclusions

In conclusion, identifying AGYW preferences for contraceptive method and service delivery characteristics is essential to developing innovative strategies to meet their unique SRH needs. Our findings directly informed the design of a DCE, which could provide valuable quantitative perspectives to guide and tailor contraceptive counseling and service delivery interventions for AGYW who want to use contraception. As the global FP community increasingly recognizes the need to shift focus from contraceptive use to contraceptive autonomy, more research is needed to understand how health systems and programs can better support AGYW in making contraceptive decisions that align with their preferences, take into account bleeding pattern and other side effect concerns, and consider their privacy needs, while reaching AGYW with quality care in accessible settings.

## Data Availability

The raw data supporting the conclusions of this article will be made available by the authors, without undue reservation.
