# Supplementary material for: Priorities for contraceptive method and service delivery attributes among adolescent girls and young women in Kenya: a qualitative study
Source: Front Reprod Health. 2024 May 7;6:1360390. doi: 10.3389/frph.2024.1360390 (PMC11107089; doi:10.3389/frph.2024.1360390)
Supplement: Supplementary file 1 [file Table1.docx]

| Candidate **Attributes** and *Levels* | Final **Attributes** and *Levels* |
| --- | --- |
| **How your periods may change**  *Periods stay the same; Periods are irregular; Periods are heavier; Periods stop* | **How your periods may change**  *Periods stay the same; Periods are irregular, but with less bleeding; Periods are heavier; Periods stop* |
| **Chance of pregnancy in 1 year**  *1/100, 10/100, 20/100* | **Chance of pregnancy in 1 year**  *1/100, 10/100, 20/100* |
| **Ability to keep method use private**  *Difficult to keep method use private; Can keep method use somewhat private; Can keep method use completely private* | **Ability to keep a method private**  *Difficult to keep method use private; Can keep method use somewhat private; Can keep method use completely private* |
| **How long a method will last**  *Using during or after sex; Use method every day; Method lasts 3 months; Method lasts at least a year* | **How long a method will last**  *Using during or after sex; Use method every day; Method lasts 3 months; Method lasts at least a year* |
| **Timing of return to fertility after stopping method**  *Immediately; within 1 month; within 6 months* | Removed |
| **Privacy during method access**  *Wait in line with other women for family planning; Wait in line with people who don’t know why you are there* | Removed |
| **Location**  Family planning clinic; pharmacy | **Location**  Clinic; Pharmacy |
| **Ability to stop method on your own**  *Can stop method on your own at any time; Requires a provider visit to stop method* | Removed |
| **How you will get information or advice about your options**  *Face-to-face with a provider; Phone-based app; Phone-based app with option for text messaging follow-up* | **How you will get information about your method**  *Face-to-face with a provider; Phone-based app; Phone-based app with option for text messaging follow-up* |
| **Cost (KSH^a^)**  *Free, 50, 200, 500* | **Cost (KSH)**  *0, 100, 200, 500* |
| ^a^ Kenyan shillings | |
